# Intellectual capital and property tax reassessment performance of local authorities: The interrelationships analysis

**DOI:** 10.3389/fpsyg.2022.1060219

**Published:** 2022-12-20

**Authors:** Asma Senawi, Atasya Osmadi

**Affiliations:** ^1^School of Housing, Building and Planning, Universiti Sains Malaysia, Penang, Malaysia; ^2^Department of Built Environment Studies and Technology, College of Built Environment, Universiti Teknologi MARA Perak Branch, Seri Iskandar, Perak, Malaysia

**Keywords:** intellectual capital, property tax, property tax assessment, assessment quality, local authorities

## Abstract

Malaysia’s property tax reassessment practices have recently revealed a previously hidden phenomenon that insufficient numbers of local authorities had undertaken a revaluation. The situation raises the question of what causes variations in property tax reassessment performance and which factors contribute to these variations. Hence, this study investigates the role of intellectual capital (IC) and its components in property tax reassessment performance among Malaysian local authorities. Data were collected using structured questionnaires from a sample of 155 officers from local authorities operating in West Malaysia. Structural equation modelling (SEM) was employed to examine the hypotheses using the SmartPLS 4.0.8.2 version of the partial least squares technique. The analysis results demonstrate that only relational capital significantly affects property tax reassessment performance, whereas human and structural capital have no equivalent relationship. Intriguingly, significant interrelationships were observed among the components of IC. The research model adds theoretical value to the discourse of organizational psychology, knowledge management, and property tax reassessment management. The significant positive relationship on relational capital resulting from this research indicates that the multiple stakeholder’s behaviours impacted reassessment work. This study offers practical managerial implications for the related parties: local authorities, public institutions and other stakeholders. The findings will change the manager’s behaviour in realizing the importance of IC and making effective strategies to improve their property tax reassessment performance.

## Introduction

The local authority plays an essential role in addressing the basic needs of all the communities in its territory. The Local Government Act, 1976 (Act 171) stipulated that each local authority has control over and responsibility for all the locations within its area for a public purpose. The Act further granted local authorities the right to impose rates on all properties within its boundary, with the state government’s approval (section 127, Act 171). Assessment rates, broadly known as property taxes, are tax levies imposed on personal property; these contribute around 60% to Malaysian local authorities’ finances (Daud et al., 2013).

Although executing property tax is generally an aim for all local authorities, their role in the policy is not always adequately understood. One key obstacle to executing property tax is periodic reassessment (Daud et al., 2013; Piracha and Moore, 2016; Agnoletti et al., 2020; Abd Rahman et al., 2021). Malaysia’s property tax reassessment practices have recently revealed a previously hidden phenomenon that most local authorities had undertaken no revaluation for almost 35 years, while only a few had the latest tone of the list for their property tax (Abd Rahman et al., 2021). The ‘tone of the list’ or ‘tone’ refers to the level of values established in a valuation list (Bond and Brown, 2017), which is generally expressed in a year form.

The above scenario portrays the inferior achievement of property tax reassessment among Malaysian local authorities and raises the questions of what causes variations in property tax reassessment performance and which factors contribute to these variations. Another query to be answered is what strategies need to be taken by managers in local authorities to be successful in this performance. Obtaining accurate answers to these questions would enable all local authorities to conduct revaluation activities efficiently. The existing situation formed the motivation for this research, which focuses on the factors associated with property tax reassessment performance. In this study, property tax reassessment performance is referred to as the local authorities’ ability to reassess property tax in a periodic cycle (as the respective legislation requires) and maintain uniformity in their assessments.

Various forms indicate and measure the organization’s performance from tangible and intangible aspects. According to Barney (1991) resource-based view (RBV) theory, the resources of an organization create a competitive advantage and contribute to its performance. The RBV theory regards firm resources, tangible or intangible, as the drivers behind competitiveness and organizational effectiveness. One intangible resource of an institution is intellectual capital, a knowledge-based resource. Intellectual capital (IC) is the sum of individual knowledge and skills, defined as the synthesis of individuals’ knowledge and capabilities that gives a firm a competitive advantage (Xu et al., 2019). A close review of the existing literature shows that staff levels of expertise, skills and education; computer software use; and the relationship with the public has become the determinants of property tax reassessment performance (Cohen et al., 2020; Massawe, 2020; Senawi et al., 2022), and these are closely linked to the concept of IC.

While IC has been identified as the primary driver of organizational success and crucial in the current knowledge economy, the literature showed that IC studies in the public sector were rarely reported, particularly in Malaysia (Buallay and Hamdan, 2019; Xu et al., 2019; Campos et al., 2020; Kamaruddin and Abeysekera, 2021). The previous research on IC primarily discusses related to the private sector while at public sector perceived in public universities (Alexander, 2018; Omowumiodeniyi, 2018; Lee and Lin, 2019; Ramírez and Tejada, 2019; Xu et al., 2019; Campos et al., 2020; Liu and Jiang, 2020). The lower IC deliberation rate in the public sector is because public managers usually pay little attention to non-financial organization resources.

Besides, RBV is generally applied in the context of overall organizational performance, and a lack of evidence has been found to engage RBV in other desired specific outcomes. This study mainly focused on one of the local authorities’ performances, known as property tax reassessment. The property tax reassessment is associated with local authorities’ financial performance as stipulated under Section 137[3] of Act 171, which is currently at a level of concern (Abd Rahman et al., 2021). Integrating property tax reassessment as a new variable with IC based on RBV’s ideology aims to advocate for better practices in property tax assessment.

Consequently, it is undoubtedly valuable to integrate IC with property tax reassessment performance to ensure local authorities undertake reassessments that follow periodic cycles, as explained further in the next section. This study advocates better practice in property tax reassessment activity by incorporating IC and its components. Besides, it will provide a reference for managers and shareholders in local authorities in making effective strategies in reassessment practice. Adopting IC will enhance property tax reassessment performance and boost the overall success of local authorities. Regular reassessment would contribute to a uniform property tax policy with substantial and heterogeneous impacts across different income groups and regions (Cao and Hu, 2016; Zhu and Dale-Johnson, 2020). Uniformity would enable the highest-quality property tax effectiveness and efficiency.

## Literature review

### Property tax

A significant factor associated with the performance of local authorities is their financial management (Pawi et al., 2011). Since property tax collection contributes around 60% of the local authorities’ financial resources, it is essential to emphasize property tax performance (Daud et al., 2013). According to data.worldbank.org (2020), Malaysia’s property tax (part of the other taxes category) contributes about 2.3% of total country revenue, increasing at about 0.3% from the preceding year. Property tax performance is measured by various elements and numerous administrative practices, including property tax base, collection, compliance, assessment, exemption, billing, and enforcement (Nyabwengi and K’Akumu, 2019).

Previous research in Malaysia primarily discussed the issues of property tax arrears, property tax non-compliance, property tax appeal procedure, and general property tax performance (Atilola et al., 2017, 2019; Mohd et al., 2018; Sahari et al., 2020; Abdullah et al., 2022). This trend is similar in other regions, especially developing countries, which set fewer observable priorities for property tax assessment (Piracha and Moore, 2016; Jashari, 2020; Carrillo et al., 2021). Besides, research in property tax assessment is immature and limited to exploring the factors related to property tax reassessment. Prior property tax reassessment research used secondary data and was conducted qualitatively, leading to theoretical and methodological gaps (Ross and Mughan, 2018; Kim et al., 2020; Abd Rahman et al., 2021). In addition, the implementation of reassessment is still inferior in practice and should not be passed over where no revaluation has been undertaken for almost 35 years by the majority of Malaysian local authorities (Abd Rahman et al., 2021). Given these points, this study focused on the performance of property tax in the reassessment context.

### Property tax reassessment

This research examines the local authorities’ performance in the context of property tax reassessment. Reassessment is “the relisting and revaluing of all property within an assessment district after finding that the original assessment is too faulty for correction through the usual procedures of review and equalization” (International Association of Assessing Officers, 2013). Malaysian property tax reassessment must be conducted once every 5 years (Section 137[3], Act 171), and the new valuation list must be prepared upon reassessment work.

**Figure 1:**
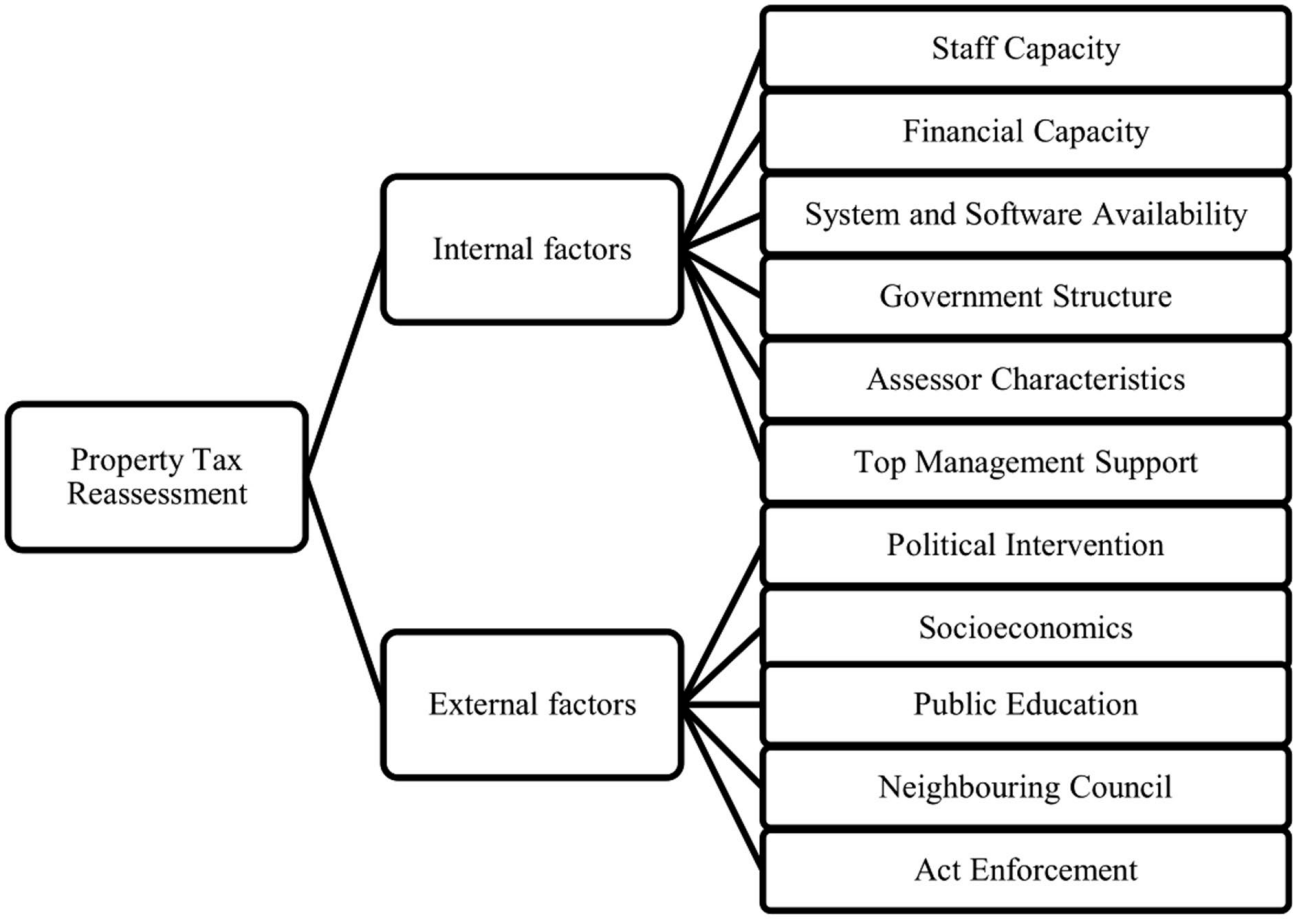
Determinants of property tax reassessment. Reproduced with permission from Senawi et al. (2022).

Whereas the existing research has rarely discussed property tax reassessment determinants, several authors have highlighted such factors in research on different contexts of property tax issues, as shown in Figure 1. The previous works can be divided into two main sub-fields: internal and external factors. The latter, which include socioeconomic conditions and political interference, are not easily controlled and lead to property tax uncertainty. Internal factors, however, are manageable and can be improved to maintain high-quality property tax reassessment performance.

To date, very few studies have examined the factors contributing to property tax reassessment activity (Senawi et al., 2022). In the literature, analyses of the determinants of property tax reassessment are primarily associated with the intangible resources of local authorities. Several reviews suggest that sufficient human resources, adequate property systems and software, good public relations and a competent assessor may contribute to the successful implementation of property tax reassessment. The current study emphasizes the association between the intangible assets of local authorities and their reassessment practices (see Figure 1). In addition, compared to the socioeconomic and political factors, intangible resources are easier to control and manage with regard to maintaining property tax performance. The latest evidence also indicates that internal determinants are more significant than external ones (Senawi et al., 2022). In addition, evidence found that one of the impediments to property tax reassessment is a lack of knowledge in preparing the paperwork for revaluation to be submitted to the state government (Abd Rahman et al., 2021).

Based on the finding on the significant relationship between intangible assets and property tax reassessment, this research is conducted to integrate IC as it is first needed to cater for this issue compared to other determinants. The knowledge asset is essential in property tax reassessment activity as a fundamental that can create value for this performance. Therefore, IC and its dimensions (human capital, structural capital and relational capital) are adopted to be the first to test their relationship with the property tax reassessment performance. The study expands the works by Senawi, Osmadi and Abd Rahman et al. (2022) to identify the actual impacts of IC on property tax reassessment performance. Using multi-scales for this study will ensure that the constructs are comprehensively measured.

### Intellectual capital and property tax reassessment performance

IC has been extensively investigated in the literature, and different perspectives on its components have been adopted. The vast majority of the studies included in this review found three main elements of IC: human capital (HC), structural/organizational capital (SC) and relational capital (RC; Ferreira and Franco, 2017; Obeidat et al., 2017; Kamaluddin and Bakar, 2019; Xu et al., 2019; Liu et al., 2020; Masoomzadeh et al., 2020; Secundo et al., 2020). The public-sector dimensions of HC refer to aptitudes for pursuing target performances, a sense of ownership and motivations that look almost identical from a private-sector perspective (Manes Rossi et al., 2016). On the other hand, the author defined SC as the procedures and routines that support decision-making, achieving objectives and handling changes. Structural capital is also known as organizational capital or internal capital since the concept covers the internal features of an organization (Kamaruddin and Abeysekera, 2013; Wang et al., 2015). In addition, RC (also known as external capital) was defined as a combination of values, relationships and acts (Manes Rossi et al., 2016).

Furthermore, a closer look at the existing literature shows that staff levels of expertise, skills and education; the use of computer software; and the relationship with the public have become the key determinant factors of property tax reassessment performance (Cohen et al., 2020; Massawe, 2020; Abd Rahman et al., 2021), and these are closely linked to the concept of IC. Previous studies have also revealed that human resources is one of the main internal factors contributing to a successful property tax revaluation (Senawi et al., 2022). The human resources of an organization is a component of IC, specifically the HC dimension. Since HC has become dominant over the other elements of IC (Bontis et al., 2015), it has been suggested that IC has a positive association with the determinant factors of property tax reassessment.

On the other hand, property tax systems and software components are linked with SC characteristics. This interconnection is supported by evidence in the literature that SC comprises hardware, software, organizational structures, patents, trademarks and other factors that support or increase employee productivity (Bontis et al., 2015; Mahmood and Mubarik, 2020). The hardware and software highlighted here refer to computers, valuation systems, land records and any related items that valuation and property management staff may have and use to support property tax reassessment activities. Recent data indicate that property land records, inventories and computer software can lead to a successful reassessment process (Senawi et al., 2022). Therefore, a close positive relationship between SC and property tax reassessment has been demonstrated.

Besides, public relations and education factors are closely related to another IC dimension, RC. Close interaction between employees of an organization and its partners has become an indicator of RC (Liu and Jiang, 2020). In the context of property tax reassessment, a closer relationship between stakeholders, governments and taxpayers is needed to make the revaluation process successful. The stakeholders and high-level government will help in the decision to conduct property tax reassessment, whereas the taxpayer’s concern is to reduce the number of objection and appeals cases, which motivates the reassessment work. Massawe (2020) recommended that continuous focus is required to ensure a high degree of public awareness of the property tax reform objectives and procedures, where the main agenda is to reassess property tax value. Other findings in the literature suggest that one property tax reform strategy would be to introduce more and better taxpayer education to increase public understanding and acceptance of rising property tax bills (Slack and Bird, 2014). Hence, the existing situation proposed that RC positively impacts property tax reassessment performance.

In conclusion, all the property tax reassessment determinants are related to the IC components, which are classified into three main dimensions: HC, SC, and RC. Although the elements of IC in property tax reassessment performance have not been directly discussed, local authorities have embraced the concept. Hence, adopting IC and its components as substitutes for certain property tax reassessment determinants is undoubtedly realistic and logical. Therefore, the IC components were adopted as independent variables for this study setting.

## Underpinning theory—the resource-based view

**Figure 2:**
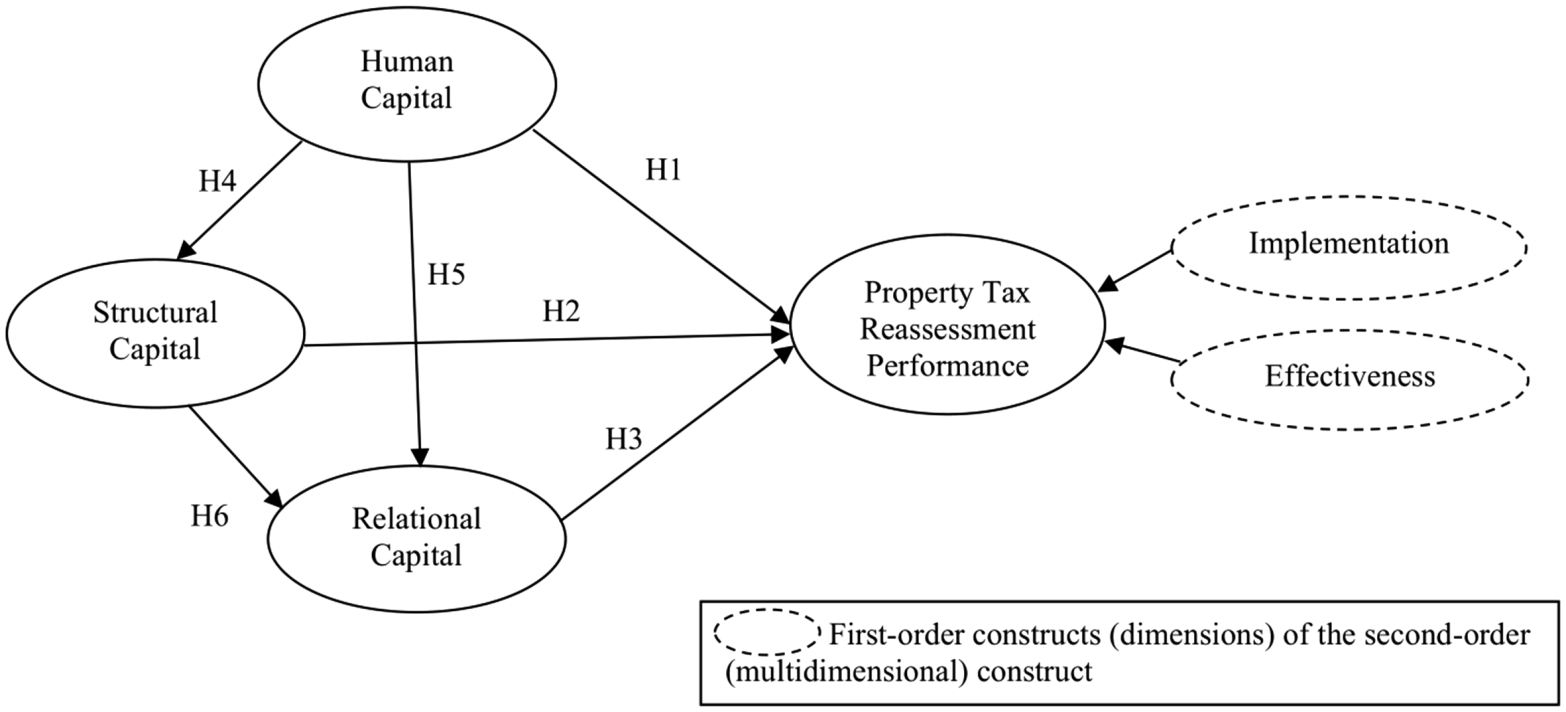
Research model.

The theory employed offers a useful understanding of the variables involved in a study. In the last two decades, the RBV has been widely accepted as one of the most potent and prominent theories for describing, explaining and predicting organizational relationships (Barney et al., 2011). In particular, it can be used to successfully explain and predict organizational performance. The RBV also explains and reviews how the performance of an organization varies, depending on the number of resources and capabilities owned by the organization (Peteraf and Barney, 2003). The resources that have been observed are invisible assets, entrepreneurship, functionally based distinctive competencies, as well as a unique combination of business experience and human resources. Since local authorities have limited resources that prominently contribute to property tax reassessment advantages, this study focuses on their intangible resources, which must be well positioned to create value and thus increase the assessment quality and property tax collection; hence, fairness among the taxpayers is ensured. For this reason, the RBV (Penrose, 1959;Wernerfelt, 1984; Barney, 1991) was chosen as the theoretical foundation for this study. The RBV theory was adopted by identifying relevant constructs for developing the research model, as shown in Figure 2.

## Hypotheses development

### Intellectual capital and property tax reassessment performance relationship

The theoretical foundation of the RBV (Wernerfelt, 1984; Barney, 1991) confirms the impact of resources and capabilities on organizational performance. Therefore, all the IC dimensions (HC, SC, and RC) were considered in this study in order to determine whether they positively impacted property tax reassessment performance. Despite the limited number of studies that have attempted to ascertain the impacts of HC, SC, and RC on property tax reassessment, sufficient empirical evidence supports the hypothesis that HC, SC, and RC have positive and significant effects on organizational performance.

Researchers have discovered that HC is an essential form of IC, and it is argued that the other two dimensions (SC and RC) originate from it (Wang et al., 2015; Farah and Abouzeid, 2017). The findings of a recent report describe how HC is positively related to organizational performance in the public sector (Kamaruddin and Abeysekera, 2014; Farah and Abouzeid, 2017; Busenan et al., 2018). Furthermore, HC also positively impacts performance in other sectors and companies, such as small and medium-sized enterprises (SMEs), manufacturing, pharmaceuticals and publicly listed companies (McDowell et al., 2018; Kweh et al., 2019; Ahmed et al., 2020; Shahwan and Fathalla, 2020). In the property tax reassessment context, research indicates that adequate and experienced human capital contributes to successful reassessment implementation (Daud et al., 2013; Kuusaana, 2015; Propheter, 2016; Eom et al., 2017; Atilola et al., 2019; Senawi et al., 2022). An organization can benefit from human capital resources through the human knowledge and experience available to it during its operations, eventually contributing to property tax reassessment performance success and enhancement.

In addition, research indicates that SC positively impacts organizational performance. Researchers have implied that SC positively impacts organizational performance in the public and private sectors (Kamaruddin and Abeysekera, 2014; McDowell et al., 2018; Ahmed et al., 2020). Adequate local authorities resources such as high-quality land records, property mapping and inventories, as well as computer software and hardware contribute to higher performance in property tax revaluation practices (Dornfest, 2008; Babawale, 2013; Dimopoulos and Moulas, 2016; Nyabwengi and K’Akumu, 2019; Awasthi et al., 2020; Massawe, 2020). The resources available in the organizations, such as up-to-date systems, and stable procedures and policies, can help them work successfully, leading to better performance in reassessment work.

Meanwhile, past research indicates that RC significantly affects organizational performance (Kamaluddin and Bakar, 2019; Yusoff et al., 2019; Masoomzadeh et al., 2020). These findings suggest that RC is also an essential contributor to property tax reassessment since it is linked to creating relationships with the public to educate them on the importance of activities that return benefits to them (Dornfest, 2008; Kuusaana, 2015; Prabhakar, 2016; Mishra et al., 2020). The relationship building with the public, government, stakeholders and other institutions will motivate them in reassessment works. After all, the findings demonstrate that the multiple dimensions of IC significantly affect an organization’s overall performance. Therefore, the authors suggested three hypotheses concerning the positive relationships between the components of IC and the property tax reassessment performance of local authorities. Hence, the connections between the IC components and the performance of property tax reassessment were hypothesized as follows;

*H1*: HC has a positive relationship with local authorities’ property tax reassessment performance.

*H2*: SC has a positive relationship with local authorities’ property tax reassessment performance.

*H3*: RC has a positive relationship with local authorities’ property tax reassessment performance.

### Interrelationships among human capital, structural capital, and relational capital

The interrelationship between the dimensions of IC has few interpretations. Recent findings indicate that the interaction between these components leads to several outcomes for an organization, like improved financial performance in the hotel industry (Sardo et al., 2018) and enhanced entrepreneurial opportunity recognition among SMEs (Rahman et al., 2022). Several studies suggest that HC needs a support system to leverage its effect on performance, and such a system is provided through SC and RC (Singla et al., 2022). Prior literature suggests that HC endorses the organizational component, an essential aspect of SC (Sardo et al., 2018). Bontis (1998) argued that without SC, IC would just be HC, and HC strongly influences SC. The employee’s competency, experience, skills and commitment will shape the organization’s systems, procedures, operations and policies, affecting property tax reassessment performance.

In addition, Singla et al. (2022) argued that RC resulted from better HC in a project performance context. Similarly, Rahman et al. (2022) examined the relationships that both HC and SC had with RC, finding these to be positive and significant in the context of employees in Omani SMEs. Rahman et al. (2022) demonstrated that HC strongly influences SC and that SC shapes the RC of SMEs in the Sultanate of Oman. Therefore, it is proposed that, with high competency and experience level, the local authorities officers influence the organizational relationship with others and actively change knowledge, leading to high property tax reassessment performance. Likewise, the stable local authorities’ procedures, operations and policies affect their relationship development with other parties such as the public, stakeholders, government, universities and private institutions. The strong relational building will enhance the performance of property tax reassessment.

In conclusion, the knowledge, expertise and skills gained by valuation staff help them to invest in information technology, good property databases and organizational development. RC is a source of successful networking, requiring knowledge, skills, expertise, a stable organization structure and state-of-the-art databases. Hence, RC was expected to enhance property tax reassessment performance by combining human and structural capital. Thus, the following hypotheses were formulated:

*H4*: HC positively influences SC.

*H5*: HC positively influences RC.

*H6*: SC positively influences RC.

## Materials and methods

### Data collection procedure and sampling

Self-administered and electronic questionnaires were used to collect data to suit the research situation and limitations. Back translation of the initial questionnaires between English and Malay was performed to ensure the accuracy and clarity of the items. Subsequently, the authors pre-tested the questionnaire using the expert judgement of academicians and practitioners in real estate and social science research. The process continued with cognitive interviews with the target respondents to establish the validity and reliability of the questionnaire. The authors arrived at the final version of the questionnaire using the pre-testing feedback to add and subtract items.

Based on the purposive sampling method, data were collected from West Malaysian local authorities, specifically from valuation officers of grades W29 to W54 in the valuation and property management departments. The W29 to W54 officers combine support, management, and professional staff who acknowledge having a high judgement on property tax matters. W29 officers are the lowest grade positioned as the head of the valuation and property management department in West Malaysian Local Authorities, whereas W54 officers are the highest. Due to different legislation and time constraints, the compiled data only reflected Peninsular Malaysia, excluding Sabah, Sarawak and the Federal Territories of Labuan. The exclusion of East Malaysia will slightly limit the research in geographical aspects.

For the final data collection, 216 questionnaires were distributed among all the local authorities in West Malaysia, and 157 questionnaires were returned for analysis. However, only 155 questionnaires were usable due to duplicate responses, as respondents answered in hard and electronic copies. The samples comprised 79 female (51%) and 76 male (49%) respondents, thus representing the genders almost equally. The statistics based on their years of service were obtained using five categories: <5 years (*n* = 30, 19%), 5–10 years (*n* = 38, 25%), 10–15 years (*n* = 39, 25%), 15–20 years (*n* = 18, 12%) and > 20 years (*n* = 30, 19%). Most respondents were of grades W29 to W36 (*n* = 118, 76%). Meanwhile, 37 (24%) respondents were of grades W41 to W54. These statistics also indicated that most of the respondents come from support staff.

### Measurement

The IC dimensions (human capital, structural capital and relational capital) were examined as independent variables of property tax reassessment performance (the dependent variable). The item measurements were developed in three steps, as in Table 1: literature review, expert judgement, and cognitive interview. The rationale for these stages is to establish content validity and reliability. During the first step, the measurements of IC were adapted from those previously used in validated research in a public organization: human capital had 25 items, structural capital had 17 items, and relational capital had 18 items (Cohen and Vlismas, 2013;Fazlagic and Szczepankiewicz, 2018; Ramírez et al., 2020). The measurement for property tax reassessment performance was developed based on previous property tax research, with five items used. The questionnaire also involves eight items for the demographic profile section.

**Table 1:** Steps in the development of the final survey.

Step	Nature of Activity	Methods	Number of Domain	Number of Items	Addition or Subtraction
I	Development of constructs and indicators	Literature review	4	73	NIL
II	Establishment of the content validity	Expert Judgement	4	52	+4 and −25
III	Establishment of the reliability	Pre-testing using cognitive interview	4	53	+1

After the expert judgement stage by 10 experts (7 academicians and 3 practitioners) in real estate and social science research, 25 items were subtracted, and four items were added. The last step during pre-testing using a cognitive interview with six actual respondents increases the total number of items to 53. Hence, the final items for the survey used 12 items for human capital (HC), 11 for structural capital (SC), 12 for relational capital (RC), 10 for property tax reassessment performance (PTRP) and eight for the demographic profile. All independent constructs use reflective indicators, as most recent literature suggests (Barrutia and Echebarria, 2021; Rahman et al., 2022; Singla et al., 2022). On the other hand, the dependent variable, PTRP, used the reflective-formative measurement model type. In this model, the lower-order constructs are reflectively measured constructs that do not share a common cause but rather form a general concept that fully mediates the influence on subsequent endogenous variables (Chin, 1998). The rationale for doing this is that the PTRP construct has two dimensions – implementation and effectiveness.

The reflective indicators of HC consist of qualifications, experience, training, skills, loyalty, creativity, motivation, and satisfaction (Cohen and Vlismas, 2013; Fazlagic and Szczepankiewicz, 2018). Besides, the items for SC subsist technical equipment, an up-to-date database, organizational structure and internal process adaptation, organizational culture, and promotional tools (Fazlagic and Szczepankiewicz, 2018; Ramírez et al., 2020). In addition, RC indicators are expressed by relations with the public, with other clients, with other public institutions, with suppliers, and with universities and images (Fazlagic and Szczepankiewicz, 2018). Lastly, PTRP indicators involve implementation and effectiveness aspects, where implementation elements include the occurrence and frequency of reassessment and the preparation to conduct a reassessment (Local Government Act, 1976; Eom et al., 2017). The effectiveness aspect incorporates the equity and uniformity of assessment, act compliance, tax collection impact, cost and tax burden (Local Government Act, 1976; Mehta and Giertz, 1996; Cornia and Walters, 2005; Eom et al., 2017; Asongu et al., 2021). In the questionnaire, the respondents were told to indicate their level of agreement with certain given statements to obtain feedback on the constructs of the research framework. Five-point and seven-point Likert-type scales from 1 (*strongly disagree*) to either 5 or 7 (*strongly agree*) were used to measure the constructs of the variables employed. Different Likert scales can be used to minimize bias in research.

### Common method variance

Common method variance (CMV) might have been a concern in this study because the independent and dependent variables were collected simultaneously from the same respondents. Following the approaches used in previous studies, both procedural and statistical remedies were performed to assess the existence of CMV. For the procedural remedies, the recommendations of Podsakoff et al. (2012) were followed, so the authors adopted a single common-method-factor approach to controlling for CMV. First, we selected four items developed by Fugate et al. (2009) that were collected in the same survey but not included in the tested model. These were used as marker indicators. Second, a method factor was created using the marker indicators as an exogenous variable to predict each endogenous construct in the model. Finally, we compared the method factor model with the baseline model and found that the significant paths in the baseline model remained significant in the method factor model.

Next, we tested the CMV using statistical remedies, as suggested by Kock and Lynn (2012) and Kock (2015), to test full collinearity. Using this method, all the variables are regressed against a common variable, and if the VIF ≤ 3.3, no bias arises from the single-source data. The analysis yielded a variance inflation factor (VIF) of less than 3.3, as shown in Table 2 below. Thus, single-source bias was not a severe issue with our data. Along this line, it was concluded that the data had no CMV issues.

**Table 2:** Full collinearity testing.

HC	SC	RC	PTRP
1.944	1.825	2.009	1.223

HC, Human Capital, SC, Structural Capital, RC, Relational Capital, PTRP, Property Tax Reassessment Performance.

## Data analysis and results

We used the SmartPLS 4.0.8.2 version of partial least squares (PLS) modelling (Ringle et al., 2022) as the statistical tool to examine the measurement and structural models since the normality assumption was not required and survey research is usually not normally distributed (Chin et al., 2003).

### Measurement model

We followed the suggestions of Anderson and Gerbing (1988) to test the developed model using a two-step approach. First, we tested the measurement model to assess the validity and reliability of the instruments used, following the guidelines of Hair et al. (2019) and Ramayah et al. (2018). Then, we ran the structural model to test our hypotheses.

For the measurement model, we assessed the loadings, average variance extracted (AVE) and composite reliability (CR). Loading values should be ≥0.5, AVE values should be ≥0.5 and CR values should be ≥0.7. As shown in Table 3, the AVE values were all higher than 0.5 and the CR values were all higher than 0.7. The loadings were also acceptable, with only 12 loadings less than 0.708 (Hair et al., 2019). For the formative second-order construct, Table 4 shows that the VIF values for implementation and effectiveness were all below the 3.33 threshold (Diamantopoulos and Siguaw, 2006). The results, therefore, did not indicate a multicollinearity problem. The two measurement models were recorded for the PTRP construct since it is a higher-order model, and the analysis used the disjoint two-stage approach. The selection for this approach is that this method shows a better parameter recovery of paths compared to the (extended) repeated indicator approach (Sarstedt et al., 2019).

**Table 3:** Measurement model for the first-order constructs.

First-Order Constructs (Reflective)	Items	Loadings	AVE	CR
Human Capital	HC1	0.638	0.601	0.947
	HC2	0.751		
	HC3	0.689		
	HC4	0.801		
	HC5	0.831		
	HC6	0.840		
	HC7	0.862		
	HC8	0.816		
	HC9	0.797		
	HC10	0.794		
	HC11	0.676		
	HC12	0.776		
Structural Capital	SC1	0.653	0.620	0.947
	SC2	0.760		
	SC3	0.758		
	SC4	0.825		
	SC5	0.886		
	SC6	0.894		
	SC7	0.840		
	SC8	0.816		
	SC9	0.780		
	SC10	0.772		
	SC11	0.634		
Relational Capital	RC1	0.698	0.563	0.939
	RC2	0.717		
	RC3	0.805		
	RC4	0.825		
	RC5	0.693		
	RC6	0.745		
	RC7	0.828		
	RC8	0.791		
	RC9	0.644		
	RC10	0.653		
	RC11	0.822		
	RC12	0.747		
Property Tax Reassessment Performance (Implementation)	IMP1	0.834	0.585	0.846
	IMP2	0.833		
	IMP3	0.792		
	IMP4	0.566		
Property Tax Reassessment Performance (Effectiveness)	EFF1	0.694	0.563	0.885
	EFF2	0.823		
	EFF3	0.681		
	EFF1	0.775		
	EFF2	0.755		
	EFF3	0.768		

**Table 4:** Measurement model for the second-order construct.

Second-Order Construct (Formative)	Indicators	Weights	VIF
Property Tax Reassessment Performance (Implementation)	IMP	0.121	1.232
Property Tax Reassessment Performance (Effectiveness)	EFF	0.942	1.232

In step 2, we assessed the discriminant validity using the HTMT criterion suggested by Henseler et al. (2015) and updated by Franke and Sarstedt (2019). The HTMT values should be ≤0.85, and the stricter criterion and the mode lenient criterion should be ≤0.90. As shown in Table 5, the HTMT values were all lower than the stricter criterion threshold of ≤0.85. We could therefore conclude that the respondents understood that the five constructs were distinct. These validity tests showed that the measurement items were valid and reliable.

**Table 5:** Discriminant validity for the first-order constructs (HTMT ratio).

Variable	1	2	3	4	5
1. Effectiveness					
2. Human Capital	0.345				
3. Implementation	0.540	0.184			
4. Relational Capital	0.407	0.662	0.299		
5. Structural Capital	0.355	0.649	0.218	0.628	

### Structural model

As Hair et al. (2017) and Cain et al. (2017) suggested, we assessed the multivariate skewness and kurtosis. The results showed that the collected data were multivariate normal for only Mardia’s multivariate skewness (*β* = 1.844, *p* < 0.001) but not for Mardia’s multivariate kurtosis (*β* = 26.509, *p* < 0.05). Thus, following the suggestion of Hair et al. (2019), we reported the path coefficients, standard errors, *t*-values, and *p*-values for the structural model using a 5,000-sample re-sample bootstrapping procedure developed by Ramayah et al. (2018). Moreover, based on the criticism of Hahn and Ang (2017) that *p*-values are not a good criterion for testing the significance of the hypothesis, the use of a combination of criteria—such as *p*-values, confidence intervals and effect sizes—was suggested. Table 6 summarizes the criteria used to test the research hypotheses.

**Table 6:** Hypotheses testing direct effects.

Hypothesis	Relationship	Std beta	Std error	t-value	p-value	BCI LL	BCI UL	f^2^	Result
H1	HC ➔ PTRP	0.016	0.115	0.141	0.444	−0.180	0.198	0.000	Not supported
H2	SC ➔ PTRP	0.095	0.104	0.910	0.182	−0.086	0.257	0.006	Not supported
H3	RC ➔ PTRP	0.353	0.093	3.803	*p* < 0.001	0.195	0.499	0.082	Supported
H4	HC ➔SC	0.616	0.059	10.503	*p* < 0.001	0.503	0.698	0.611	Supported
H5	HC ➔ RC	0.423	0.086	4.938	*p* < 0.001	0.270	0.554	0.207	Supported
H6	SC ➔ RC	0.331	0.092	3.604	*p* < 0.001	0.182	0.481	0.127	Supported

We used a 95% confidence interval with a bootstrapping of 5,000.

First, we tested the effect of the three predictors on PTRP, and the *R*^2^ was 0.182 (*Q*^2^ = 0.052), which showed that all three predictors explained 18.2% of the variance in PTRP. HC (*β* = 0.016, *p* = 0.444), SC (*β* = 0.095, *p* = 0.182) and RC (*β* = 0.353, p < 0.001) were all positively related to PTRP, with only RC significant, so only H3 was supported, and H1 and H2 were rejected. Next, we tested the effect of HC on SC, and the *R*^2^ was 0.379 (*Q*^2^ = 0.364), which indicates that HC explained 37.9% of the variance in SC. The relationship of HC with SC is significant with *β* = 0.616, p < 0.001, thus supporting H4. Lastly, we tested the effects of HC and SC on RC, with the *R*^2^ of 0.462 (*Q*^2^ = 0.379) indicating that HC and SC explained 46.2% of the RC variance. The findings indicate that HC and SC influence RC significantly with *β* = 0.423, *p* < 0.001 and *β* = 0.331, *p* < 0.001, respectively, thus supporting H5 and H6.

Besides, rather than we reported the statistical significance (p-values), substantive significance (effect size) was also disclosed. Referring to Hair et al. (2019), *f*^2^ explain how the removal of a certain predictor construct affects an endogenous construct’s *R*^2^ value. As a rule of thumb, values higher than 0.02, 0.15 and 0.35 depict small, medium and large f^2^ effect sizes (Cohen, 1988). From Table 6, RC indicates a small effect in producing the *R*^2^ for PTRP. However, HC and SC do not predict the property tax reassessment performance. In addition, the results indicate that HC has a substantial effect in producing the *R*^2^ for SC. Moreover, the results show that HC has a moderate effect compared to SC, with a small effect in producing the *R*^2^ for RC.

Finally, we assessed the predictive relevance of the model through the blindfolding procedure. The predictive sample reuse technique, popularly known as Stone–Geisser’s *Q*^2^, can be applied as a criterion for predictive relevance. Henseler et al. (2009) accentuated this measure to assess the research model’s predictability. Based on the blindfolding procedure, the results indicate that the *Q*^2^ values for PTRP (*Q*^2^ = 0.052), SC (*Q*^2^ = 0.364), and RC (*Q*^2^ = 0.379) are more than 0, suggesting that the model has sufficient predictive relevance. For reference, values of 0.02, 0.15, and 0.35 indicate that an exogenous construct has a small, medium or large predictive relevance for a particular endogenous construct (Hair et al., 2017).

## Discussion

The findings and analysis offer new insights into the relationship of local authorities’ property tax reassessments with IC and its components (HC, SC, and RC). The interrelationships between the components of IC and property tax reassessment performance were tested. The findings revealed that only RC has a significant positive relationship with property tax reassessment performance, while surprisingly, SC and HC do not play an instrumental role. In addition, the interrelationship between IC elements also indicates a significant result where HC influences the SC and RC, and likewise, SC influences RC. The result concludes that the basic theory of RBV can be applied in managing property tax reassessment with the integration of IC components by prioritizing RC development. The development of RC indeed needs leverage from HC and SC. These results further confirmed the attitude of local authorities’ managers in making effective property tax reassessment strategies by integrating IC components.

In most studies, human capital has been demonstrated to play an influential role in organizational performance. In the property tax reassessment context, Abd Rahman et al. (2021) found that staff capacity was an impediment when conducting revaluation, which implies a significant impact. However, recent findings related to HC in another area found that HC did not directly affect project performance (Singla et al., 2022). This research found an insignificant relationship, possibly due to the nature of property tax reassessment activities. The result shows that employee experience, skills, competencies and commitments do not influence the property tax revaluation performance. In West Malaysia, reassessment is conducted in-house and outsourced, so the aid of private valuer competencies will lead to a successful revaluation process. The local authorities generally will assess the small holdings due to a lack of staff, and they hire a private valuer to assess larger properties. A local authority may depend on the appointed valuer’s aptitude rather than their human resources. However, this scenario is not applied in all local authorities as some will fully utilize their internal resources to conduct a revaluation. Another reason is that public organizations in Malaysia have not yet fully developed a knowledge-based economy, which requires more comprehensive knowledge, skills and experience.

Meanwhile, the results show another unsupported outcome, the insignificant effect of structural capital on property tax reassessment performance. The vital role of structural capital has also been noted in past studies of both public and private organizations (Kamaruddin and Abeysekera, 2014; McDowell et al., 2018; Ahmed et al., 2020). Structural capital - an organization’s infrastructure, systems, policies and procedures (Bontis, 1998)—makes an institution capable and enhances its competitive advantages. Unexpectedly, this did not happen in the property tax reassessment context. The local authorities’ support systems, operations, procedures and policies do not impact reassessment activity. The main reason is connected to HC, whereby outsourced resources need to be established when local authorities hire private organizations to conduct a reassessment. When outsourcing revaluation work, local authorities will use support from external resources to integrate with their existing resources available. This interpretation needs further clarification as not all West Malaysian local authorities will outsource their reassessment works. Further judgement is that property tax reassessment requires different type of structural capital, perhaps innovative systems, databases and procedures.

On the other hand, relational capital was found to have a positive and significant relationship with property tax reassessment performance. The finding is in line with previous research, which asserted that collaboration with the public, stakeholders and other external parties is required in property tax reform (Slack and Bird, 2014; Massawe, 2020). From the context of other performances, massive evidence has suggested that relational capital positively influences organizational performance, thus supporting this result (Ahmed et al., 2020; Torre et al., 2020; Hanifah et al., 2021). As the only knowledge asset dimension that influences property tax reassessment, RC has become an essential criterion for improving taxpayer education and public understanding in terms of accepting rising property tax bills. The situation will motivate local authorities to conduct property tax reassessment performance.

Furthermore, their relationship with other public institutions like their neighbors and universities leads to an active exchange of knowledge and experiences. The transfer of best practices with them will encourage better performance in property tax reassessment. Local authorities can use expertise from the universities to help them with reassessment and learn from other local authorities which had successfully conducted property tax revaluation. Besides, a strong relationship with the top level of government is needed since approval from the state government is required before implementing a reassessment. The financial aid or incentive for reassessment activities can be gathered from this relationship building, bringing better achievement in revaluation performance. The abovementioned situation reveals that the multiple stakeholders’ behaviors contribute to reassessment work. The excellent performance in property tax reassessment comes from the managers’ decisions, along with other groups within and outside the organization.

Alternatively, structural and human capital significantly influence relational capital, evidenced by two positive relationships. Despite the insignificant effects of HC and SC on property tax reassessment performance, they play a vital role when each has a positive relationship with RC. The results indicated the indirect effects of HC and SC on reassessment performance, reconfirming the earlier reported interrelationships between the three organizational elements of IC (Bontis, 1998). In addition, Sardo et al. (2018) and Rahman et al. (2022) found that HC and SC played instrumental roles in regard to RC among SMEs. The latest evidence related to project performance also indicates that HC has an indirect impact mediated through SC and RC (Singla et al., 2022). Hence, these findings also remarking the mediating role of RC in the human capital and property tax reassessment performance link and the structural capital and property tax reassessment performance relationship. The skills, knowledge and expertise of valuation staff improve the relationship between local authorities, society and stakeholders. Along similar lines, the local authority’s systems, procedures and policies help them to establish connections with the public and other institutions.

## Conclusion

### Theoretical implications

The research model adds theoretical value to the discourse of organizational psychology, knowledge management, and property tax reassessment management. Firstly, the theory of organizational psychology was added by highlighting local authorities’ attitude to succeed in property tax reassessment from a knowledge assets perspective. The study addressed the vital part of the literature gap by considering the aspect of organizational behavior in conducting reassessment work which reveals that various stakeholders (managers, staff, public, government, universities, and suppliers) are significantly involved in this performance. This aspect will add value to the psychology theory among local authority managers in implementing property tax policies. Alongside all the stakeholders, managers need to focus on other resources in local authorities: their human resources and organizational capital.

Second, this research significantly contributes to the body of knowledge and enriches understanding of the role of human, relational and structural capital in improving property tax reassessment performance. The study contributes to the theory of RBV by identifying the intangible resources needed for a property tax reassessment and how it could work. Even though IC research is not entirely new, it is diverse when it relates to IC in the context of local authorities’ property tax reassessment performance. To the best of our knowledge, this study is the first to reveal the relationships between the components of IC and organizational performance in the property tax reassessment context. The findings also confirm the interlinkages between these intangible assets and their direct and indirect impacts on property tax reassessment performance. The authors conclude that undertaking revaluation activities may be useless without the integrated support of HC, SC, and RC. The study also confirms the indirect effects of HC and SC on property tax reassessment performance.

Lastly, this study focused on the success factors related to property tax assessment, which are rarely studied in the literature. The study contributes to the theory by providing a conceptual framework of property tax management that examines the performance of property tax reassessment through the lenses of knowledge assets. Despite the other factors, intangible assets (HC, SC, and RC) confirmed having a significant impact and became one of the determinants in this performance. The local authorities need to consider these aspects to be successful in property tax reassessment. Therefore, the study provides valuable information for researchers, academics and local authorities in IC and property tax research.

### Practical implications

The research framework provides a roadmap for managers and stakeholders in local government to understand the workable links between IC and its dimensions in the context of property tax reassessment. Local authorities may add value to the property tax assessment aspect of their property tax policies, which may also help to reflect their knowledge of assets, actual property tax assessment quality, and overall property tax performance. The performance of a property tax reassessment greatly depends on building relationships such as a good affair with the public, government, universities, other agencies and suppliers, as well as actively exchanging knowledge. Therefore, it is in the best interests of external parties and local authorities to maintain healthy work relations.

A property tax reassessment activity involves the public and a higher level of government. The public and the local authorities must have a smooth relationship. Maintaining healthy relations with the public and with government agencies, understanding their requirements, and building trust are essential for implementing a property tax reassessment. At the same time, the local authorities must ensure a healthy working relationship among their staff and a stable organizational structure. As suggested, the benefits of relationship building cannot be achieved without developing HC and SC. Local authorities must establish people and organizations that focus on relationship building so that the knowledge and skills of the valuation staff, as well as the institution’s systems and procedures, can be used, which would ideally result in successful reassessment activities. The strategy must revolve around developing RC and using these resources to leverage the effects of HC and SC on property tax reassessment performance.

### Limitations and future directions

The authors developed a model explaining the interrelationships of IC and its impact on property tax reassessment performance. This probe appears to be limited in its scope to design a conceptual framework based on the literature gaps and theoretical backdrops. The other limitations of this study are question order and acquiescence bias, as there is no randomization of questions, and all items are positive. Future research into the proposed IC and property tax reassessment model could empirically explore, examine and validate the model presented in this study in different industry sectors and economies. Different social settings are required to establish and increase the generalisability of the findings and confirm or refute the theoretical relationships between the concepts.

## Data availability statement

The raw data supporting the conclusions of this article will be made available by the authors, without undue reservation.

## Author contributions

AS: guarantor of the integrity of the entire study, study concept and design, literature research, data analysis, statistical analysis and manuscript preparation. AO: study concept and design, manuscript preparation and manuscript editing. All authors contributed to the article and approved the submitted version.

## Funding

Ministry of Higher Education Malaysia funded this research for the Fundamental Research Grant Scheme with Project Code: FRGS/1/2020/SS0/USM/02/30 and Universiti Sains Malaysia.

## Conflict of interest

The authors declare that the research was conducted in the absence of any commercial or financial relationships that could be construed as a potential conflict of interest.

## Publisher’s note

All claims expressed in this article are solely those of the authors and do not necessarily represent those of their affiliated organizations, or those of the publisher, the editors and the reviewers. Any product that may be evaluated in this article, or claim that may be made by its manufacturer, is not guaranteed or endorsed by the publisher.
